# Whole-blood transcriptome profiling reveals signatures of metformin and its therapeutic response

**DOI:** 10.1371/journal.pone.0237400

**Published:** 2020-08-11

**Authors:** Monta Briviba, Laura Ansone, Ivars Silamikelis, Vita Rovite, Ilze Elbere, Laila Silamikele, Ineta Kalnina, Davids Fridmanis, Jelizaveta Sokolovska, Ilze Konrade, Valdis Pirags, Janis Klovins

**Affiliations:** 1 Latvian Biomedical Research and Study Centre, Riga, Latvia; 2 Faculty of Medicine, University of Latvia, Riga, Latvia; 3 Faculty of Medicine, Riga Stradins University, Riga, Latvia; Chinese Academy of Sciences, CHINA

## Abstract

Metformin, a biguanide agent, is the first-line treatment for type 2 diabetes mellitus due to its glucose-lowering effect. Despite its wide application in the treatment of multiple health conditions, the glycemic response to metformin is highly variable, emphasizing the need for reliable biomarkers. We chose the RNA-Seq-based comparative transcriptomics approach to evaluate the systemic effect of metformin and highlight potential predictive biomarkers of metformin response in drug-naïve volunteers with type 2 diabetes *in vivo*. The longitudinal blood-derived transcriptome analysis revealed metformin-induced differential expression of novel and previously described genes involved in cholesterol homeostasis (*SLC46A1* and *LRP1)*, cancer development *(CYP1B1*, *STAB1*, *CCR2*, *TMEM176B)*, and immune responses (*CD14*, *CD163*) after administration of metformin for three months. We demonstrate for the first time a transcriptome-based molecular discrimination between metformin responders (delta HbA1c ≥ 1% or 12.6 mmol/mol) and non-responders (delta HbA1c < 1% or 12.6 mmol/mol), that is determined by expression levels of 56 genes, explaining 13.9% of the variance in the therapeutic efficacy of the drug. Moreover, we found a significant upregulation of *IRS2* gene (log_2_FC 0.89) in responders compared to non-responders before the use of metformin. Finally, we provide evidence for the mitochondrial respiratory complex I as one of the factors related to the high variability of the therapeutic response to metformin in patients with type 2 diabetes mellitus.

## Introduction

Diabetes mellitus is a chronic disease affecting approximately 463 million people worldwide, which is nearly 9.3% of the global population [1]. Type 2 diabetes mellitus (T2DM) is the most common type of diabetes accounting for approximately 90% of all cases. The persistent hyperglycemia and insulin resistance, a characteristic of T2DM patients, is associated with an increased risk of serious microvascular and macrovascular complications, including nephropathy, retinopathy, neuropathy, myocardial infarction, and stroke, which may be reduced by early initiation of antidiabetic therapy [2–4]. Metformin is the first-line medication for treating hyperglycemia in T2DM with beneficial effects in the treatment of multiple non-diabetes related conditions, such as polycystic ovary syndrome, cancer, and neurodegenerative disorders [5–7]. Despite the pleiotropic effects of the drug, the variable efficacy and gastrointestinal side-effects observed cause a significant non-compliance and discontinuation of the therapy, justifying a need for studies exploring molecular mechanisms of metformin action, and biomarkers predicting both treatment response and tolerance of the drug [8].

The mechanism of metformin action is generally considered to involve modulation of the activity of mitochondrial complex I, activation of 5′ AMP-activated protein kinase (AMPK)-dependent mechanisms, and increased AMP concentrations, though some controversy remains since multiple studies are providing evidence for other indirect mechanisms, such as the significant contribution of the gut microbiome underlying the glucose-lowering effect of the drug [9–11].

RNA sequencing (RNA-Seq) is the state-of-the-art approach that may be used to profile drug response and efficacy biomarkers [12, 13]. So far, transcriptome datasets obtained from cell cultures and tissue samples of animal models are extensively used in studies describing molecular mechanisms of metformin concerning various conditions, nevertheless, longitudinal data of *in vivo* studies in humans are still lacking. RNA-Seq has revealed various novel effects and therapeutic targets of metformin, such as enrichment of the transcriptional regulator forkhead box O3a (*FOXO3a*) in primary human fibroblasts [14], upregulation of activating transcription factor 3 (*ATF3*) in primary human hepatocytes [15], downregulation of cell division control protein 42 homolog (*CDC42*) in breast cancer cells [16], upregulation of krüppel-like factor 4 (*KLF4*) resulting in suppressed endothelial dysfunction [17], and even modulated alternative splicing in embryonic stem cells [18]. Moreover, multiple animal-based studies have reported metformin-specific signatures in gene expression profiles of rat arteries and mice epididymal fat, liver and muscle tissue, nevertheless, they still do not explain many beneficial effects of the drug [19–21].

Although the relatively high proportion (>30%) of patients failing to achieve glycemic control during metformin therapy has been partially explained by the contribution of genetic inheritance (allelic variants of organic cation transporters *OCT1*, *OCT2*, etc.) [22–24], recent studies report that heterogeneity of metformin response may be both patient and cell type-specific, suggesting the presence of yet unknown, non-genetic and selective manifestations of the drug [25, 26]. The main objective of the study was to assess the systemic effect of metformin in T2DM patients and reveal potential biomarkers for accurate prediction of its therapeutic efficacy. We have previously reported direct evidence of the effects of metformin on the immediate and strong transcriptome changes in whole-blood samples of healthy subjects, though we considered the diabetic state as a significant confounding factor, therefore the study was continued in a well-characterized, prospective T2DM patient cohort with prolonged observational time, providing much wider applicability of the study results [27]. We believe that our strategy will promote the development of biomarker-based approaches for monitoring treatment outcomes and early identification of metformin responders, moving towards precision medicine.

## Materials and methods

### Study design

The study was conducted within the framework of the ongoing observational, prospective and longitudinal study OPTIMED, which has been implemented since 2010 in collaboration with endocrinologists and general practitioners from the leading health care centers in Latvia, ensuring recruitment of newly diagnosed drug-naïve patients with ICD-10 diagnosis code E11 and follow-up data collection. Written informed consent was obtained from every participant after full explanation of the purpose and nature of all procedures used before their inclusion in the study, and the study protocol was approved by Central Medical Ethics Committee of Latvia (No. 01–29.1/22) and Committee of Ethics in Pauls Stradins Clinical University Hospital (No.3000610 - 18L). The research was conducted in accordance with The Code of Ethics of the World Medical Association (Declaration of Helsinki amended in Fortaleza, Brazil, October 2013) and The Convention for the protection of Human Rights and Dignity of the Human Being with regard to the Application of Biology and Medicine: Convention on Human Rights and Biomedicine. Management of patient recruitment, collection of samples and associated clinical data was ensured by Latvian Biomedical Research and Study Centre’s core facility Genome Centre and the Genome Database of the Latvian Population following their standard procedures [28].

In total, 17 patients of European descent fulfilling the following inclusion criteria were enrolled: (1a) newly diagnosed type 2 diabetes mellitus (ICD-10 code E11) requiring oral antidiabetic therapy, or (1b) previously diagnosed type 2 diabetes mellitus but no oral antidiabetic therapy or insulin has been used for the last three years, or (1c) newly diagnosed type 2 diabetes mellitus and intensive insulin therapy initiated in a hospital for acute glycemic normalization; (2) the patient is not currently involved and is not planning to enroll in clinical trials during the OPTIMED study; (3) the patient has attained 18 years of age; (4) the patient is not pregnant at the time of application; (5) the patient meets the criteria for the diagnosis of type 2 diabetes mellitus: (a) fasting blood glucose level ≥ 7 mmol/l, (b) a blood glucose level ≥ 11.1 mmol/l for a two-hour glucose tolerance test with 75 g intake. The exclusion criteria of the study were as follows: (1) the patient is receiving oral antidiabetic therapy on a regular basis or has received the therapy during the last three years; (2) the patient is receiving insulin therapy at the time of application; (3) the patient is pregnant. According to the observational study design, the randomization procedure was not performed and metformin monotherapy (medication with metformin hydrochloride as the only active ingredient) was prescribed for each study participant by an endocrinologist for at least three months regardless of the research objectives. The drug manufacturer and dosage of metformin (varied from 850 mg to 2000 mg per day) were chosen by endocrinologists based on clinical experience, patient’s health status, and manifestations of the disease. Enrollment in the study did not affect the choice of treatment strategy made by endocrinologists. Blood tests (e.g. measures of ALT, creatinine levels, HbA1c) were performed in a certified clinical laboratory before the administration of metformin and after metformin therapy for three months to evaluate general hematological and biochemical parameters and eligibility of the subjects (Table 1). Blood samples for RNA-Seq were collected at the same time points, hereinafter referred to as M0 (before administration of metformin) and M3m (after three months long metformin course). Considering the high inter-individual variability expected in RNA-Seq data, longitudinally repeated measurements were chosen as the most suitable approach for global gene expression analysis.

**Table 1:** Characteristics of the study group.

Characteristic	Value
Female/ male, n (%)	11 (64.7%) / 6 (35.3%)
Mean age (years) ± SD	61.12± 9.57
Mean BMI ± SD	34.94 ± 4.70
Mean ALAT ± SD, μkat/L	0.74 ± 0.51
Mean creatinine ± SD, μmol/l	63.25 ± 12.60
Mean triglycerides ± SD, mmol/l	2.51 ± 1.86
HbA1c level before the therapy ± SD, mmol/mol	60 ± 14
HbA1c level after 3 months of metformin therapy ± SD, mmol/mol	46 ± 6

BMI, body mass index; SD, standard deviation; ALAT, alanine aminotransferase; HbA1c, glycated hemoglobin.

HbA1c measurements were made for all participants at two consecutive time points analogous to the blood collection for RNA extraction (M0 and M3m). Study participants were further stratified in two subgroups based on metformin response, which was defined according to metformin-induced alterations in glycated hemoglobin (HbA1c) levels comparing measurements made in the time points M0 and M3m: responders (delta HbA1c ≥ 1% or 12.6 mmol/mol), non-responders (delta HbA1c < 1% or 12.6 mmol/mol) [29].

### Sample processing and RNA sequencing

For RNA isolation, 3 ml of whole blood were collected in Tempus™ Blood RNA Tubes (Thermo Fisher Scientific, USA) and further processed using Tempus™ Spin RNA Isolation Kit (Thermo Fisher Scientific, USA) according to manufacturer’s instructions. The quantity and quality of extracted RNA and prepared libraries were determined by Qubit Fluorometer (Thermo Fisher Scientific, USA) and Agilent 2100 Bioanalyzer systems (Agilent, USA), respectively. The integrity of RNA was evaluated by RNA integrity number (RIN) within the Agilent 2100 Bioanalyzer system (Agilent, USA). For depletion of ribosomal RNA 500 ng of total RNA from each sample were processed using Low Input RiboMinus™ Eukaryote System v2 (Thermo Fisher Scientific, USA). Complementary DNA library preparation was performed with Ion Total RNA-Seq Kit v2 (Thermo Fisher Scientific, USA). Ion Proton™ System (Post-Light™ Ion Semiconductor Sequencing, Thermo Fisher Scientific, USA) and Ion PI™ Chip (Thermo Fisher Scientific, USA) was used for 200-base-read single-end sequencing, following the manufacturer’s instructions. Since the shot-gun RNA-Seq is considered to be the most accurate and desirable method for the quantification of the individual transcript and gene expression, additional methods for technical validation were not applied in this study [30].

### Data analysis

Trimmomatic 0.36 was used for read trimming applying window size 5 and quality threshold of 10. After trimming reads had to have a minimum length of 30 bp and an average quality of 10 to be included in subsequent analyses. Sequencing reads were mapped against human reference genome GRCh38 release 90 and per-gene read counts were calculated with STAR (v.2.5.3a.). STAR (v.2.5.3a.) outputs read quantification per gene while performing read mapping. The reads were quantified if they match only one gene. The obtained read counts were then normalized using trimmed mean normalization implemented in Bioconductor package edgeR in R (v.3.5.3). Differentially expressed genes (DEGs) were estimated using the Likelihood ratio test with added observation weights to reduce the influence of outliers, and sva (Surrogate Variable Analysis) package in R (v.3.5.3) was used for removing batch effects [31]. In order to evaluate metformin-induced alterations in the transcriptome profile (comparison of samples M3m vs M0), each sample was set as a factor considering longitudinal study design. When comparing responders against non-responders in each time point separately, sequencing run and baseline (M0) HbA1c levels were considered as covariates. FilterByExpr function was applied for gene filtering in edgeR, taking into account the sample library sizes [32]. Multiple testing correction was implemented using the Benjamini-Hochberg procedure and differential expression of the genes was determined using a false discovery rate (FDR) < 0.05 cutoff, regardless of the log_2_ fold change of expression for each gene [33]. In order to identify key genes determining the metformin response, Partial least squares discriminant analysis (PLS-DA) was performed implemented in the mixOmics package of R (v.3.5.3). CPM values (obtained in edgeR and adjusted for the impact of sequencing run and baseline HbA1c levels) were used in PLS-DA. Key genes contributing to a separation of patients in both metformin response groups were identified by using a cutoff of variable importance of projection (VIP) score >1 obtained from PLS-DA [34]. Association between HbA1c levels and the log CPM expression values of each mitochondrial gene was performed with multiple linear regression using lm function in R (v.3.5.3). Sex and body mass index were included in the model to account for their potential confounding.

Genes showing the p-value <0.05 for differential expression were further used in the functional analysis. Gene Ontology (GO) terms and Kyoto Encyclopedia of Genes and Genomes (KEGG) pathways were adopted as the functional terms. GO enrichment analysis was performed with R package Goseq (v.1.38.0), and KEGG pathway enrichment analysis was done using an online software Database for Annotation, Visualization and Integrated Discovery (DAVID) 6.8., the threshold value of enrichment was selected by a P-value <0.05 [35, 36].

Heat maps were constructed with Matplotlib⁠ and SciPy⁠. Hierarchical clustering with average linkage method implemented in SciPy was used for the clustering of genes according to their differences in CPM values [37, 38]. Statistical analysis of anthropometric measures and biochemical data was performed in R (v3.5.3.) by applying the Wilcoxon rank-sum test and Pearson's chi-squared test with a p-value threshold < 0.05.

## Results

### Identification of metformin-induced differential expression of genes in blood cells

We characterized the transcriptome profiles of whole-blood samples obtained from 17 drug-naïve T2D patients (characteristics of patients provided in Table 1) before any antidiabetic therapy (M0) and after three months of metformin monotherapy (M3m) by RNA-Seq technique to detect sustained transcriptional alterations in blood cells at the diabetic state due to the administration of the drug. The median of read counts per sample produced by RNA sequencing was 21.1 (IQR = 6.3) and 87.9% of the reads (median = 18.2; IQR = 5.1) were further mapped to the human reference genome.

**Fig 1:**
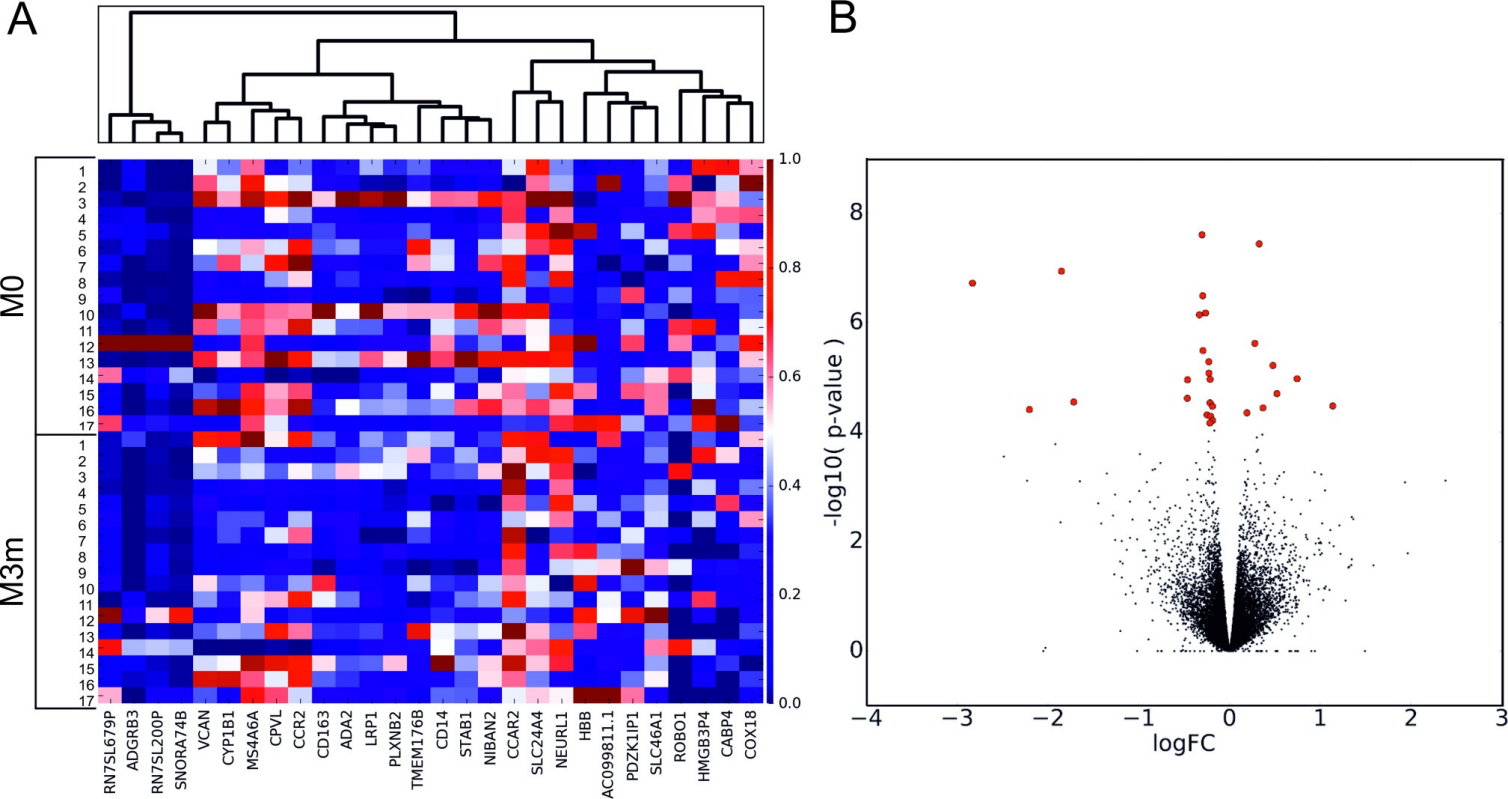
Metformin-induced alterations of gene expression profiles. (A)—Heat map and hierarchical clustering of 28 DEGs identified. Each row corresponds to one subject in the respective time-point and each column represents a DEG. Normalized sequence read counts were rescaled to lie in the range [0,1] and further used to estimate the difference between the gene expression levels in two time-points. DEGs with analogous expression values were clustered at the column level. (B)—Volcano plot showing the distribution of gene expression in the analyzed contrast. Significance versus log_2_ fold change is plotted on the y and x axes, respectively, calculated using likelihood ratio test and edgeR. Red dots represent the significant DEGs (FDR < 0.05), black dots–non-significant genes. M0 –time point of blood collection before administration of metformin; M3m –time point of blood collection after metformin therapy for three months.

Differential expression analysis performed by comparing transcriptome profiles of blood samples collected after metformin administration for three months against samples collected before the use of any antidiabetic therapy (M3m vs M0) revealed 28 DEGs (FDR < 0.05) from the pool of 9992 transcripts identified in total. Out of them, 20 genes were significantly down-regulated and 8 genes showed significant up-regulation after administration of metformin (Fig 1, Table 2, S1 Fig).

**Table 2:** List of differentially expressed genes due to administration of metformin, ranked by log_2_ fold change.

Gene symbol	Full name	log_2_FC	FDR
*ROBO1*	Roundabout guidance receptor 1	-2.83	7.54E-04
*ADGRB3*	Adhesion G protein-coupled receptor B3	-2.2	2.67E-02
*CABP4*	Calcium binding protein 4	-1.85	6.08E-04
*HMGB3P4*	High mobility group box 3 pseudogene 4	-1.72	2.45E-02
*NEURL1*	Neuralized E3 Ubiquitin Protein Ligase 1	-0.46	2.26E-02
*COX18*	Cytochrome C Oxidase Assembly Factor	-0.46	1.17E-02
*NIBAN2*	Niban apoptosis regulator 2	-0.33	1.64E-03
*STAB1*	Stabilin 1	-0.3	2.89E-04
*CPVL*	Carboxypeptidase vitellogenic like	-0.29	1.03E-03
*TMEM176B*	Transmembrane protein 176B	-0.29	5.72E-03
*SLC24A4*	Solute Carrier Family 24 Member 4	-0.26	1.64E-03
*CYP1B1*	Cytochrome P450 family 1 subfamily B member 1	-0.25	3.10E-02
*LRP1*	LDL receptor related protein 1	-0.23	8.23E-03
*CCR2*	C-C motif chemokine receptor 2	-0.23	1.11E-02
*CD14*	CD14 molecule	-0.22	3.88E-02
*VCAN*	Versican	-0.21	2.45E-02
*MS4A6A*	Membrane Spanning 4-Domains A6A	-0.21	1.17E-02
*CD163*	CD163 molecule	-0.21	3.15E-02
*PLXNB2*	Plexin B2	-0.19	2.57E-02
*ADA2*	Adenosine deaminase 2	-0.19	3.62E-02
*CCAR2*	Cell cycle and apoptosis regulator 2	0.19	2.95E-02
*HBB* ^†^	Hemoglobin subunit beta	0.28	4.77E-03
*PDZK1IP1* ^†^	PDZK1-interacting protein 1	0.33	2.89E-04
*SNORA74B*	Small nucleolar RNA, H/ACA box 74B	0.37	2.61E-02
*RN7SL679P* ^†^	RNA, 7SL, cytoplasmic 679, pseudogene	0.48	8.77E-03
*RN7SL200P*	RNA, 7SL, cytoplasmic 200, pseudogene	0.52	1.96E-02
*AC099811*.*1*	AC099811.2 (novel transcript, sense intronic to STAT5B)	0.74	1.17E-02
*SLC46A1*	Solute carrier family 46 member 1	1.14	2.57E-02

Log_2_FC, log_2_ fold change; FDR, false discovery rate.

^†^Genes showing significant differential expression due to the administration of metformin also in healthy individuals [27].

### Functional enrichment characteristic to metformin therapy

In order to describe the implementation of metformin-modulated transcriptome profiles in cell signaling pathways and core biologic functions, the KEGG pathway and GO enrichment analysis was performed. Over-representation of biological pathways (e.g. amino sugar and nucleotide sugar metabolism, antigen processing and presentation) and GO Terms (e.g. helper T cell chemotaxis and lipoprotein particle receptor activity) related to energy metabolism, immune responses and lipid metabolism were observed, see S1 and S2 Tables for the complete list of GO terms and enriched KEGG pathways.

### Determining genes involved in differential metformin responsiveness

Considering the potential contribution of distinct molecular mechanisms mediating variable metformin response, all participants were stratified in two efficacy groups, according to metformin-induced changes in their HbA1c levels (responders: delta HbA1c ≥1% or 12.6 mmol/mol; non-responders: delta HbA1c <1% or 12.6 mmol/mol) (Table 3).

**Table 3:** Characteristics of responders and non-responders.

Characteristic	Responders (n = 10)	Non-responders (n = 7)	P-value
Female/ male, n (%)	6(60.00%)/4(40.00%)	5(71.43%)/2(28.57%)	1.00E+00
Mean age (years) ± SD^†^	61.14±11.35	61.10±8.77	8.84E-01
Mean BMI ± SD^†^	33.70±4.50	35.81±5.12	2.61E-01
Mean ALAT ± SD, μkat/L^†^	0.91±0.70	0.61±0.27	4.91E-01
Mean creatinine ± SD, μmol/l^†^	59.57±13.35	66.11±12.75	2.23E-01
Mean triglycerides ± SD, mmol/l^†^	3.65±2.57	1.75±0.67	8.78E-02
HbA1c level before the therapy ± SD, mmol/mol^†^	76±15	48±4	4.48E-03
HbA1c level after 3 months of metformin therapy ± SD, mmol/mol	48±7	45±4	5.57E-01

BMI—body mass index; SD—standard deviation; ALAT—alanine aminotransferase; HbA1c - glycated hemoglobin.

^†^Measured before the administration of metformin (M0).

**Fig 2:**
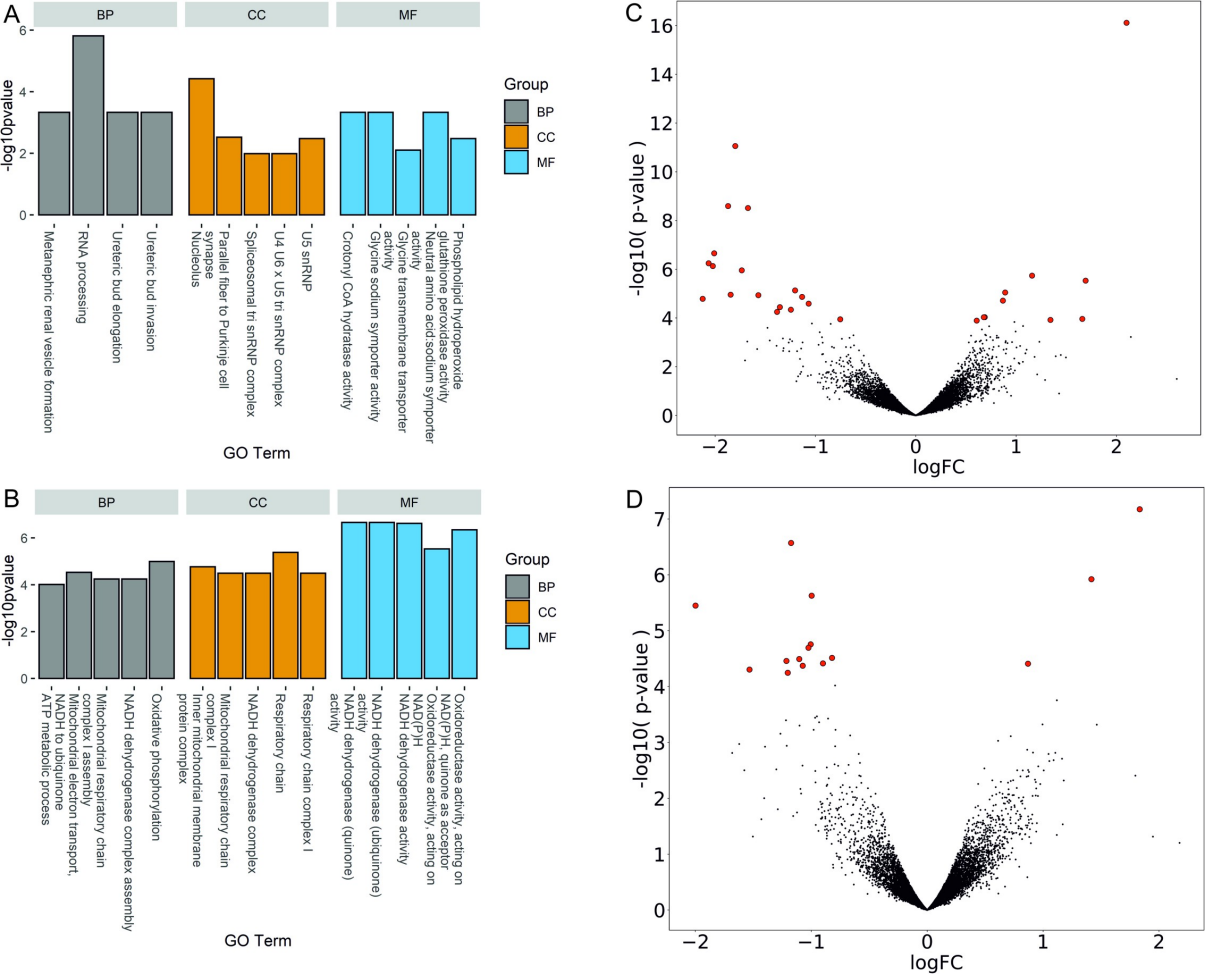
Differentially expressed genes and their representation in Gene Ontologies. Bar plots showing the top 5 enriched Gene Ontology terms in the comparison of responders against non-responders before administration of metformin (A) and three months after metformin therapy (B). Volcano plot represents the distribution of gene expression comparing responders against non-responders before administration of metformin (C) and three months after metformin therapy (D). Significance versus log_2_ fold change is plotted on the y and x axes, respectively, calculated using likelihood ratio test and edgeR. Red dots represent the significant DEGs (FDR < 0.05), black dots–non-significant genes. BP–biological process; CC–cellular component; MF–molecular function.

Genes associated with variable metformin responsiveness were determined by differential gene expression analysis comparing the number of RNA-Seq reads from responders against non-responders in each time point of blood collection (M0 and M3m) separately. In total, 27 significant DEGs were identified contrasting responders against non-responders before the administration of metformin (M0). We observed a notable portion of downregulated genes coding for small nucleolar RNAs and upregulated insulin receptor substrate 2 (*IRS2*) gene in responders, revealing distinctive features between response groups already before the use of any antidiabetic therapy (Fig 2C), (Table 4).

**Table 4:** List of differentially expressed genes comparing responders against non-responders before administration of metformin.

Gene symbol	Full name	log_2_FC	FDR
*RNU5A-1*	RNA, U5A small nuclear 1	-2.12	1.07E-02
*SNORA20*	Small nucleolar RNA, H/ACA box 20	-2.06	9.85E-04
*SNORD82*	Small nucleolar RNA, C/D box 82	-2.02	1.11E-03
*SNORA5C*	Small nucleolar RNA, H/ACA box 5C	-2.01	4.63E-04
*SNORA28*	Small nucleolar RNA, H/ACA box 28	-1.87	8.00E-06
*RNU5B-1*	RNA, U5B small nuclear 1	-1.84	8.63E-03
*SNORD20*	Small nucleolar RNA, C/D box 20	-1.8	4.58E-08
*RNY4*	RNA, Ro60-associated Y4	-1.73	1.44E-03
*SNORD90*	Small nucleolar RNA, C/D box 90	-1.67	8.00E-06
*SNORA75*	Small nucleolar RNA, H/ACA box 75	-1.57	8.63E-03
*SNORA74B*	Small nucleolar RNA, H/ACA box 74B	-1.38	2.80E-02
*SNORD91A*	Small nucleolar RNA, C/D box 91A	-1.35	1.98E-02
*SNORA37*	Small nucleolar RNA, H/ACA box 37	-1.24	2.39E-02
*SNORA66*	Small nucleolar RNA, H/ACA box 66	-1.2	7.00E-03
*SNORA14B*	Small nucleolar RNA, H/ACA box 14B	-1.13	9.45E-03
*CLC*	Charcot-Leyden crystal galectin	-1.07	1.49E-02
*RALGPS2* ^†^	Ral GEF with PH domain and SH3 binding motif 2	-0.75	4.74E-02
*GPX4*	Glutathione peroxidase 4	0.61	4.95E-02
*PCMTD2*	Protein-L-isoaspartate (D-aspartate) O-methyltransferase domain containing 2	0.68	4.23E-02
*MARCH2*	Membrane associated ring-CH-type finger 2	0.69	4.23E-02
*CDYL*	Chromodomain Y like	0.87	1.19E-02
*IRS2*	Insulin receptor substrate 2	0.89	7.81E-03
*SIRPG*	Signal regulatory protein gamma	1.16	2.13E-03
*SLC6A9*	Solute carrier family 6 member 9	1.34	4.83E-02
*FMN1* ^†^	Formin 1	1.66	4.74E-02
*HEBP1* ^†^	Heme binding protein 1	1.69	3.06E-03
*CHI3L1*	Chitinase 3 like 1	2.1	8.03E-13

Log2FC, log2 fold change; FDR, false discovery rate.

^†^Genes showing significant differential expression comparing responders against non-responders also after metformin therapy for 3 months.

**Fig 3:**
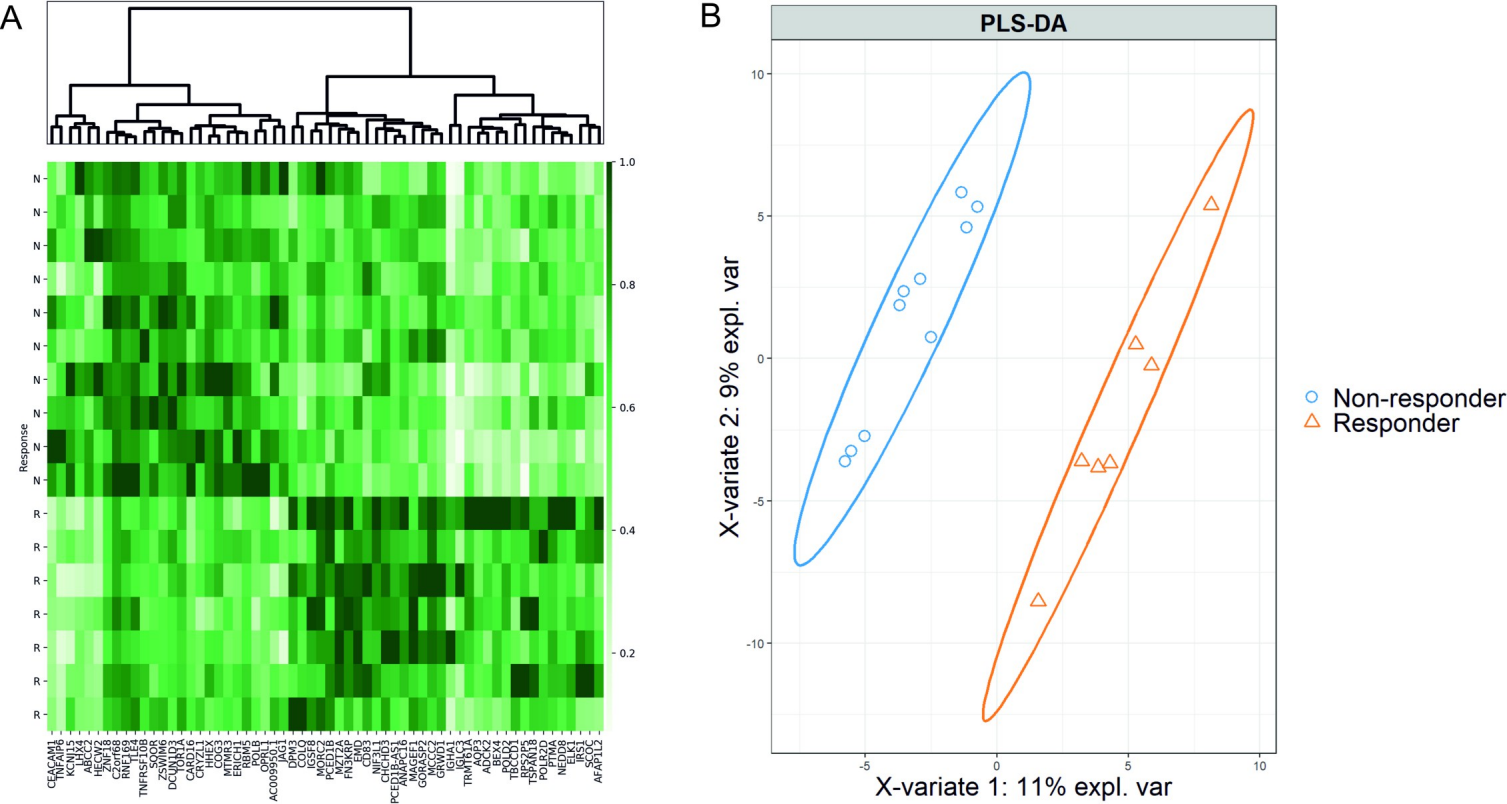
The partial least-square discriminant analysis of the RNA-Seq data obtained before the administration of metformin. (A) Heat map and hierarchical clustering of 56 key genes contributing the most to the patient separation in metformin response groups, selected by VIP score threshold >1 from PLS-DA. Each row corresponds to one subject (N: non-responder; R: responder) and each column represents a gene. Normalized sequence read counts were rescaled to lie in the range [0,1], genes with analogous expression values were clustered at the column level. (B) PLS-DA plot of RNA-Seq data showing clear transcriptome-based discrimination of patients with different metformin responses. Each point represents the transcriptome signature of one patient, the confidence level is set to 95% for ellipses. The separation of samples in the PLS-DA model is based on latent variables (X-variate 1 on and X-variate 2 on x and y axes, respectively). T2DM patients with different metformin responses are projected into distinct clusters indicating the difference in their transcriptome profiles.

PLS-DA was applied to understand whether patients could be clustered based on their gene expression profiles before the administration of metformin. PLS-DA revealed pronounced discrimination of samples, based on the obtained transcriptome profiles, explaining 13.9% of the variance in total (11% of variance explained by latent variable 1 and 2.9% of variance explained by latent variable 2) (Fig 3B). In total, 56 discriminatory genes showing the strongest separation of different metformin response groups were identified, based on variable importance for projection (VIP>1) value in two latent variables (components), generated by PLS-DA (Fig 3A). See S5 Table for the full list and VIP values of discriminatory genes.

Comparison of gene expression profiles between metformin responders and non-responders after the use of metformin for three months (M3m) showed differential expression of 15 genes (12 up-regulated, 3 down-regulated), including 5 mitochondrial genes, which may be associated with mechanisms underlying variable efficacy of the drug (Table 5) (Fig 2D). Moreover, a multiple linear regression model revealed a significant association between the expression of two out of five mitochondrial genes and HbA1c levels after metformin therapy for three months (*MT-ND4*, p-value = 0.047; *MT-ND4L*, p-value = 0.032) (S7 Table).

**Table 5:** List of differentially expressed genes comparing responders against non-responders after metformin therapy for three months.

Gene symbol	Full name	log_2_FC	FDR
*S100P*	S100 calcium binding protein P	-2.00	7.54E-03
*FP671120*.*7*	Novel transcript, similar to YY1 associated myogenesis RNA 1 YAM1	-1.53	3.77E-02
*PAX5*	Paired box 5	-1.21	3.45E-02
*TNFRSF13C*	TNF receptor superfamily member 13C	-1.20	4.01E-02
*IGHM*	Immunoglobulin heavy constant mu	-1.17	1.43E-03
*MT-ND6*	Mitochondrially encoded NADH: ubiquinone oxidoreductase core subunit 6	-1.10	3.45E-02
*MT-ND4L*	Mitochondrially encoded NADH: ubiquinone oxidoreductase core subunit 4L	-1.07	3.46E-02
*MT-ATP6*	Mitochondrially encoded ATP synthase membrane subunit 6	-1.02	3.07E-02
*MT-ND4*	Mitochondrially encoded NADH: ubiquinone oxidoreductase core subunit 4	-1.00	3.07E-02
*MT-ND2*	Mitochondrially encoded NADH: ubiquinone oxidoreductase core subunit 2	-1.00	6.27E-03
*CD79A*	CD79a molecule	-0.90	3.45E-02
*RALGPS2* ^†^	Ral GEF with PH domain and SH3 binding motif 2	-0.82	3.45E-02
*WARS*	Tryptophanyl-trna synthetase	0.87	3.45E-02
*HEBP1* ^†^	Heme binding protein 1	1.42	4.23E-03
*FMN1* ^†^	Formin 1	1.83	7.12E-04

Log2FC, log2 fold change; FDR, false discovery rate.

^†^Genes showing significant differential expression comparing responders against non-responders also before administration of metformin.

### Biological functions contributing to metformin responsiveness

GO analysis was performed for each list of DEGs (responders against non-responders at time points M0 and M3m separately). RNA processing and negative regulation of fatty acid transport was revealed among the enriched biological processes using DEG list obtained by comparison of responders against non-responders before administration of metformin (Fig 2A). Contrasting responders against non-responders after three months of metformin use identified enrichment of GO terms strongly related to the mitochondrial activity (e.g. mitochondrial respiratory chain complex I, ATP metabolic process) (Fig 2B). See S3 and S4 Tables for the full list of enriched GO terms identified. Although KEGG pathway analysis of DEG list, obtained from the baseline analysis (M0), did not reveal any significantly enriched cell signaling pathway, comparison of responders against non-responders after three months of metformin use showed differential expression of 5 mitochondrial genes (*MT-ATP6*, *MT-ND2*, *MT-ND4*, *MT-ND4L*, *MT-ND6*). All of these genes provided a significant enrichment of the following pathways: hsa00190: Oxidative phosphorylation (p-value = 4.47E-06), hsa05012: Parkinson's disease (p-value = 5.81E-06) and hsa01100: Metabolic pathways (p-value = 2.18E-02).

## Discussion

The results of our study showed metformin-specific signatures in blood cell transcriptome profiles associated with some of its well-known properties: the ability to improve energy metabolism, influence immune responses, and inhibit cancer progression. This study has demonstrated a gene expression-based molecular discrimination between metformin responders and non-responders, and suggested that mitochondrial respiratory complex I may be associated with metformin efficacy. To the best of our knowledge, this is the first longitudinal study focusing on metformin-induced transcriptional alterations of drug-naïve T2DM patients *in vivo*.

Our study revealed metformin-induced differential expression of genes involved in cholesterol homeostasis, such as solute carrier family 46 member 1 *(SLC46A1*), which is involved in the intestinal folate absorption affecting plasma high-density lipoprotein levels [39], and lipoprotein receptor-related protein 1 (*LRP1*), a crucial protein for cholesterol uptake. Metformin-induced reduction of hepatic *LRP1* expression level has been reported before [40], though here we report a similar effect in blood cells for the first time. Metformin-induced differential expression of both genes may serve as a contributing factor for the cholesterol-lowering effect of the drug. In our data, the same mechanism was supported by the enrichment of lipoprotein particle receptor activity among identified GO terms.

In addition, we found significant downregulation of multiple cancer-related genes coding for cytochrome P450 1B1 (*CYP1B1*), C-C chemokine receptor type 2 (*CCR2*), stabilin-1 (*STAB1*) and transmembrane protein 176B (*TMEM176B*). Some of the observed alterations have been previously identified in different tissue types, such as the downregulation of *CCR2*, which has been explained by the ability of metformin to block M2-like polarization of tumor-associated macrophages providing the anti-metastatic effect of the drug [41], or downregulation of *CYP1B1* in breast cancer cells where due to its crucial role in estrogen metabolism, metformin has been suggested as a potential chemopreventive agent against carcinogenesis [42]. Multiple studies have reported that aberrant expression of *STAB1* is related to the tumor progression in various types of cancer, serving as a potential molecular target for cancer therapy [43, 44]. Similarly, elevated protein levels of TMEM176B are detected in multiple malignancies, moreover, inhibition of TMEM176B has already been proved to promote CD8^+^ T cell-mediated tumor growth control, enhancing the therapeutic efficacy of cancers [45, 46]. Therefore, the observed downregulation of both, *STAB1* and *TMEM176B* may highlight novel players in the anti-cancer activity of metformin.

Finally, the enrichment of antigen processing and presentation pathway and significantly reduced expression of genes coding for the cluster of differentiation 14 (*CD14*), which was already proved to be differentially expressed in metformin-treated monocyte cells, and the cluster of differentiation 163 (CD163) [47], a scavenger receptor which has been previously associated with insulin resistance in patients with T2DM, altogether may explain well-known participation of metformin in the inflammatory and immune responses [48*]*.

According to our previous study, there are only three genes (*HBB*, *PDZK1IP1*, and *RN7SL679P*) showing metformin-induced differential expression in blood cells obtained from both, T2DM patients and healthy volunteers. Although both studies were longitudinal, the duration of the therapy (7 days for healthy volunteers and 3 months for T2DM patients) and the dose of metformin (850 mg twice a day for healthy volunteers and variable dose in the T2DM patient group), differed between both studies, which may explain the observed variability of metformin effects on the blood cell transcriptome profiles among both study groups. Moreover, the general health status including the presence of T2DM may serve as the major confounding factor to modulate the cell metabolism and effects of metformin in the patient group compared to healthy individuals [49].

One of the main findings of our study was the clear, transcriptome-based discrimination of study subjects into metformin responders and non-responders before the administration of any anti-diabetic therapy. Differential expression analysis before the use of metformin revealed significant upregulation of *IRS2* gene coding for insulin receptor-2 (log_2_FC 0.89) in responders compared to non-responders. So far, multiple studies have demonstrated the ability of metformin to activate the hepatic insulin receptor and the IRS2/PI3K/Akt pathway resulting in reduced insulin resistance and increased glucose uptake [50, 51]. The results of our study suggest that the activity of *IRS2* in blood cells prior to the administration of metformin may be related to the efficacy of the therapy. Nevertheless, this hypothesis must be tested in a larger longitudinal cohort.

PLS-DA analysis also provided strong evidence for transcriptome-based patient stratification which was mainly explained by a single latent variable (component) and defined by expression of 56 key genes (VIP>1). We found carcinoembryonic antigen-related cell adhesion molecule 1 (*CEACAM1*), Insulin receptor substrate 1 (*IRS1*), *ABCC2* gene coding for multidrug resistance protein 2 and *IGHA1* gene coding for the constant segment of immunoglobulin A heavy chain among the marker genes determining the distribution of patients in metformin response groups. So far, there are no studies reporting the role of multidrug resistance protein 2 in metformin efficacy, nevertheless, its homolog mitochondrial multidrug resistance protein 1 has been already associated with resistance to metformin in human malignant mesothelioma cells [52]. Interestingly, *CEACAM1* is a mediator of insulin clearance in the liver [53] and *IRS1* is playing a key role in glucose homeostasis [54], though no reports were linking their activity with metformin efficacy so far. In our previous studies, we have already reported the contribution of IgA, the most abundant intestinal antibody shaping the gut microbiome composition, in metformin action [27], and here we suggest a potential implication of IgA in microbiome-mediated metformin response [55]. Together these genes showed notable patient segregation in metformin response groups also when visualized in CPM-based heat map, therefore they may be considered as candidates for further studies of biomarkers for metformin response.

The comparison of blood cell transcriptome profiles from responders with transcripts from non-responders after the metformin therapy for three months revealed significant downregulation of mitochondrial genes (*MT-ATP6*, *MT-ND2*, *MT-ND4*, *MT-ND4L*, *MT-ND6*). Furthermore, *MT-ND4* and *MT-ND4L* also showed a positive association with HbA1c levels according to the multiple regression model. Metformin directly acts on mitochondria and limits respiration by inhibiting mitochondrial respiratory-chain complex 1 (NADH: ubiquinone oxidoreductase), which is among the top targets of the drug, and it also catalyzes oxidative phosphorylation in mammalian mitochondria [56, 57]. Thus, the observed downregulation of genes coding for NADH: ubiquinone oxidoreductase core subunits in responders and enrichment of oxidative phosphorylation seems rational. These data suggest altered mitochondrial complex I activity as one of the mechanisms linked to the variability of metformin response.

There are a few limitations in this study that could be addressed by future research. First, a small sample size due to the essential inclusion criterion of drug-naïve T2DM patients. Second, prolonged observation time (three months) that provides an advantage to evaluate long-term metformin effects, while causing an issue of possible accumulation of uncontrollable factors affecting gene expression and masking the true effects of the drug. Third, the lack of control arm allowing the elimination of these confounding factors. Nevertheless, we believe that RNA-Seq, a highly sensitive and accurate method for gene expression, together with the repeated measures within the longitudinal study design and strictly defined inclusion criteria ensures the absence of potential influence of other anti-diabetic therapies and improves the validity of our results.

In conclusion, the current study applying RNA-Seq for the discovery of transcriptional effects of metformin in drug-naïve T2DM patients provided detailed insight into potential molecular mechanisms underlying well-known beneficial effects of metformin. However, since there are no data confirming the accumulation of metformin in other blood cells than erythrocytes [58], it seems reasonable to assume that the observed effects of metformin on peripheral blood cells most likely are indirect and reflects the systemic consequence of the therapy. Nevertheless, we suggest that blood-derived transcriptome profiles may be used for evaluation of therapeutic efficacy and specific genes may be further applied in the development of biomarkers for metformin response.

## Supporting information

S1 Table Gene ontology analysis for the list of differentially expressed genes identified by comparing M3m samples with M0 samples. (XLSX)

S2 Table KEGG pathway enrichment analysis for the list of differentially expressed genes identified by comparing M3m samples with M0 samples. (XLSX)

S3 Table Gene ontology analysis for the list of differentially expressed genes identified by comparing responders against non-responders before administration of metformin (M0). (XLSX)

S4 Table Gene ontology analysis for the list of differentially expressed genes identified by comparing responders against non-responders after administration of metformin for 3 months (M3m). (XLSX)

S5 Table The variable importance of projection score for key genes contributing the most to the patient separation in metformin response groups before the use of any antidiabetic therapy. (XLSX)

S6 Table Summary of sequencing statistics. (XLSX)

S7 Table Association between HbA1c and gene expression levels obtained by multiple linear regression model after metformin therapy for three months. (XLSX)

S1 Fig Violin plots showing the log_2_ fold change of 28 differentially expressed genes after metformin use. Each dot represents the log2 fold change for a particular gene of one study subject. The shape of the plot represents the distribution of the data obtained using kernel density estimate and Scott's rule for bandwidth selection.
(TIFF)

S2 Fig CONSORT flowchart of the observational prospective study. (TIFF)
