# Supplementary figures and images for: Whole-blood transcriptome profiling reveals signatures of metformin and its therapeutic response
Source: PLoS One. 2020 Aug 11;15(8):e0237400. doi: 10.1371/journal.pone.0237400 (PMC7418999; doi:10.1371/journal.pone.0237400)

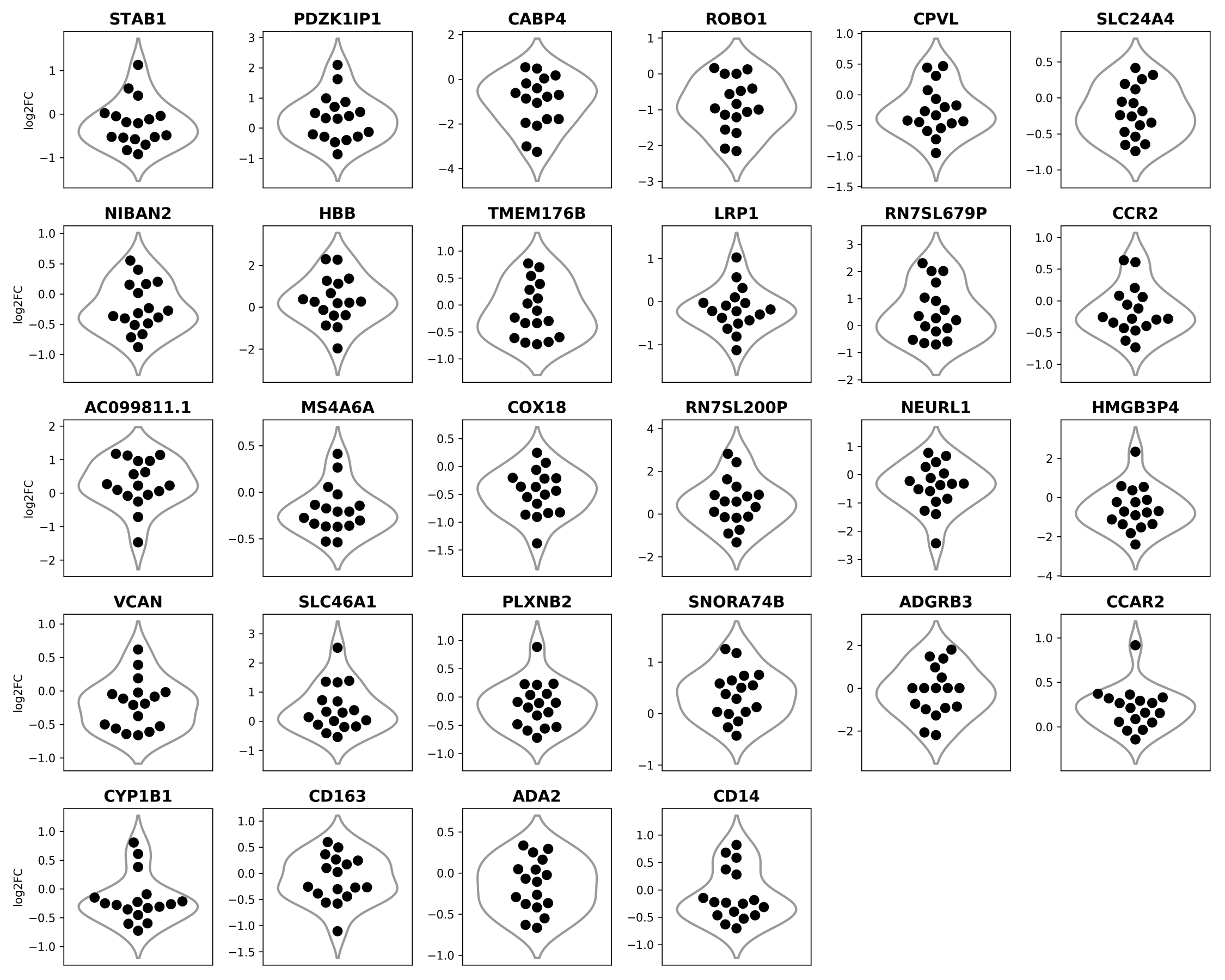

Supplement: S1 Fig — Each dot represents the log2 fold change for a particular gene of one study subject. The shape of the plot represents the distribution of the data obtained using kernel density estimate and Scott's rule for bandwidth selection. (TIFF) [file pone.0237400.s008.tiff]

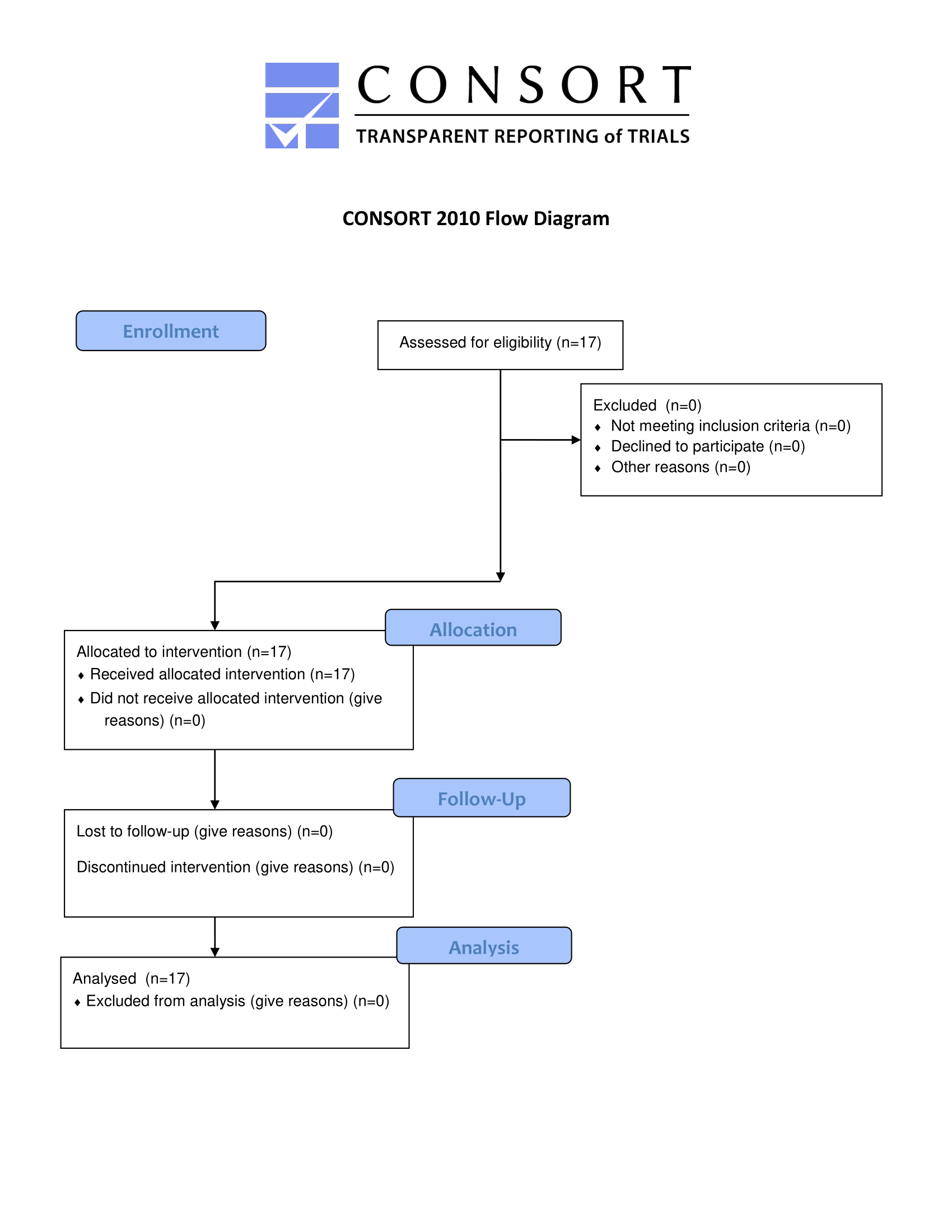

Supplement: S2 Fig — (TIFF) [file pone.0237400.s009.tiff]
